# Photodynamic Therapy-Mediated Immune Responses in Three-Dimensional Tumor Models

**DOI:** 10.3390/ijms222312618

**Published:** 2021-11-23

**Authors:** Nkune Williams Nkune, Nokuphila Winifred Nompumelelo Simelane, Hanieh Montaseri, Heidi Abrahamse

**Affiliations:** Laser Research Centre, Faculty of Health Sciences, University of Johannesburg, P.O. Box 17011, Johannesburg 2028, South Africa; 217078898@student.uj.ac.za (N.W.N.); 201904217@student.uj.ac.za (N.W.N.S.); haniehm@uj.ac.za (H.M.)

**Keywords:** photodynamic therapy, innate immunity, adaptive immunity, 3D tumor models, immunomodulation, nanotechnology

## Abstract

Photodynamic therapy (PDT) is a promising non-invasive phototherapeutic approach for cancer therapy that can eliminate local tumor cells and produce systemic antitumor immune responses. In recent years, significant efforts have been made in developing strategies to further investigate the immune mechanisms triggered by PDT. The majority of in vitro experimental models still rely on the two-dimensional (2D) cell cultures that do not mimic a three-dimensional (3D) cellular environment in the human body, such as cellular heterogeneity, nutrient gradient, growth mechanisms, and the interaction between cells as well as the extracellular matrix (ECM) and therapeutic resistance to anticancer treatments. In addition, in vivo animal studies are highly expensive and time consuming, which may also show physiological discrepancies between animals and humans. In this sense, there is growing interest in the utilization of 3D tumor models, since they precisely mimic different features of solid tumors. This review summarizes the characteristics and techniques for 3D tumor model generation. Furthermore, we provide an overview of innate and adaptive immune responses induced by PDT in several in vitro and in vivo tumor models. Future perspectives are highlighted for further enhancing PDT immune responses as well as ideal experimental models for antitumor immune response studies.

## 1. Introduction

**Figure 1:**
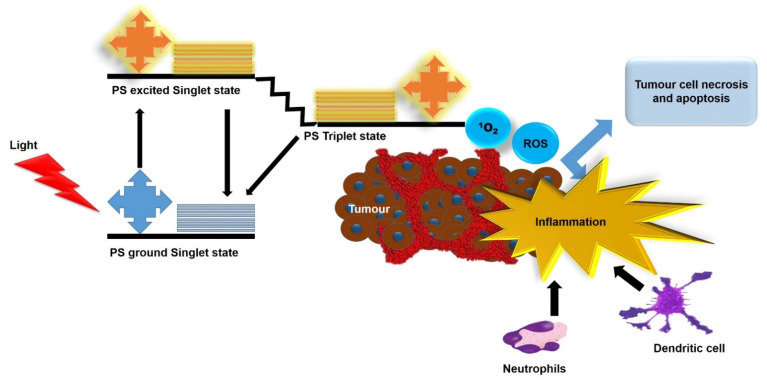
Antitumor mechanisms induced by PDT.

Photodynamic therapy (PDT) is a cancer modality that combines three essential components of a photosensitizer (PS), harmless light, and molecular oxygen [1]. It is based on the accumulation of a PS in pathological tissues, which can generate highly cytotoxic reactive oxygen species (ROS) upon its activation with a specific wavelength of light [2]. PDT presents unique advantages such as the selective uptake of PSs by tumor tissues, localized light exposure to the affected site, non-invasiveness feature, and simple procedure. Additionally, it endows low toxicity and high efficacy, with no drug resistance [3,4]. As shown in Figure 1, ROS generated by PDT can directly destruct the vasculature of the tumor by induction of apoptosis and/or necrosis, resulting in oxygen and nutrient depletion in the tumor [5]. As a result of this photodamage to the tumor and its microenvironment, a robust acute inflammatory response is produced at the tumor site [4,5]. The acute inflammatory response following PDT stimulates the immune system and causes the infiltration of host innate immune cells, which clear damaged cells in the treated area [5,6]. In a later stage, an adaptive immune memory may occur, allowing for a systemic response that can inhibit tumor recurrence and metastases in the long run [6].

When the immune monitoring function becomes dysfunctional, tumor cells can continue to grow and form malignant tumors [6,7]. Additionally, tumor cells can evade immune system barriers via multiple mechanisms, which is the main cause of the low clinical efficacy of the most antitumor treatments [8]. Generally, immunosuppressive cells and molecules counteract any antitumor effects in the body [9]. Depending on the influence of immunoregulatory factors in the tumor microenvironment, some tumors are less immunogenic and do not trigger any specific immune responses [7]. The low expression of transporter of antigenic peptide and major histocompatibility complex (MHC) molecules normally hinders antigen processing and presentation mechanisms, which in turn inhibits specific immune responses [7]. PDT can circumvent this dysfunction via the initiation of immunogenic tumor cell death modes, essentially immunogenic apoptosis and necrosis [6]. An innate immune response can be initiated by the exposure or release of danger stimuli from damaged cancer cells, which is known as damage-associated molecular patterns (DAMPs) [4,6]. These DAMPs alone or in combination with tumor antigens can be identified by antigen-presenting cells (APCs), which may trigger an adaptive immune response against the tumor [6].

Although the antitumor immune effects of PDT have been thoroughly investigated, there is a dire need for more relevant models capable of recapitulating the heterogeneity and the microenvironment of the in vivo human tumor, thus allowing for more predictive in vitro evaluation of PDT-induced antitumor immunity [4]. As traditional two-dimensional (2D) cell cultures lack cell–cell interactions and the essential tumor microenvironment responsible for tumor signaling and drug response, significant discrepancies have been reported when comparing the outcomes to in vivo experiments [10]. Thus, in vivo experiments have been extensively conducted, using immune-suppressed animal models that may not fully reflect what happens in humans [11]. However, animal-based models harbor tumors that have been artificially generated in a short period. Moreover, immune cell subsets and receptor–ligand pairs, which are imperative for tumor cell recognition and antitumor immune responses, are often different from their human counterparts [10,11]. In this context, three-dimensional (3D) tumor spheroids have been proposed as excellent culture models to reduce the number of animals used and possibly replace cumbersome and expensive animal models. Herein, 3D cell culture methods are described together with their advantages and disadvantages. Furthermore, we discussed PDT-mediated immune responses in various in vitro and in vivo tumor models. The latest advances in PDT-induced antitumor immunity for improved PDT immune response outcomes are also highlighted.

## 2. Comparison of 2D and 3D Tumor Models

**Figure 2:**
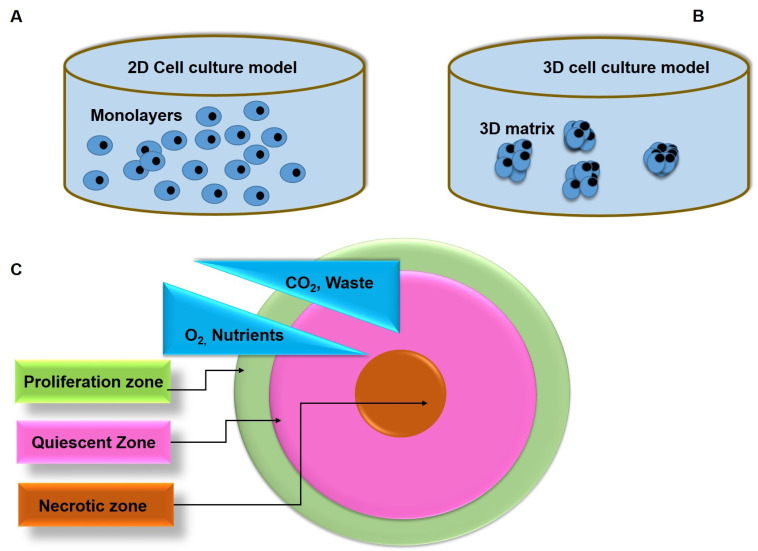
Schematic illustration of conventional 2D monolayer and 3D cell culture models. (**A**) Conventional 2D mono cell culture; (**B**) Three-dimensional (3D) cell culture modes; (**C**) Three-dimensional (3D) spheroid with an internal layered cell distribution. The regions are the proliferation zone (outer layer), quiescent zone (middle), and necrotic zone (innermost).

Cell culture is an indispensable tool in the preclinical drug development process [12]. Two-dimensional (2D) cell culture models present several advantages such as cost-effectiveness and simplicity in terms of preparation, maintenance, and monitoring, allowing for amenable microscopic and molecular investigations [13]. However, 2D cultures rely on cells adhering to the host flat surface, normally a flask or plate, which does not mimic the 3D architecture of tumor tissues (Figure 2A) [14]. Furthermore, the cells are exposed to a relatively steady and uniform source of oxygen, nutrients, and growth factors, preventing them from resembling the in the vivo tumor microenvironment (TME) [13,14]. For example, cancer-associated fibroblasts (CAFs), tumor-associated macrophages (TAMs), endothelial cells (ECs), and cancer-associated adipose (CAA) are key components of breast cancer progression and carcinogenesis that compromise the tumor microenvironment [15]. Therefore, 2D cell culture models cannot predict drug efficacy in a clinical setting and often lead to unwanted toxicity and low success rates in drug validation and approval procedures [12].

In recent years, 3D tumor cultures have emerged as excellent cancer models, which can bridge between preclinical and clinical studies for better evaluation of novel anticancer drugs [9]. Spheroids as a type of 3D tumor cultures closely resemble solid tumors in vivo as they exhibit different aspects of a solid tumor cellular heterogeneity, oxygen and nutrients, cell–cell signaling, cell–cell contacts, growth kinetics, and resistance to anticancer agents (Figure 2B) [9,16]. The spheroid structure shows an internal layered cell distribution similar to solid tumors (Figure 2C) [17]. Within this structure, various forms of cells, such as proliferating, quiescent, apoptotic, hypoxic, and necrotic cells are established [12,17]. Accordingly, due to access to oxygen and nutrients from the culture media, proliferating cells are located in the outer layer of the spheroids and resemble cancer cells close to the capillaries in vivo [12,18]. Conversely, the middle layer is characterized by quiescent cells because cell metabolism decreases progressively as the distance from the spheroid periphery increases [17]. Cells at the core of spheroids typically undergo necrosis due to the deprivation of oxygen, growth factors, and nutrients, as well as the accumulation of waste products [19]. Therefore, it is logical to theorize that the cellular processes of these cultures are relevant at this juncture, due to the morphological features, cellular interactions, and heterogeneity of 3D cell cultures [20].

Several studies have compared 2D and 3D cell cultures in terms of morphology, cell survival, proliferation, differentiation, cellular responses to stimuli, cell–cell signaling mechanisms, cell rigidity, metastatic and invasive characteristics of tumor cells into adjacent tissues, together with angiogenesis stimulation and immune checkpoints evasion, response to therapeutics, gene expression, and general functional properties (Table 1).

## 3. Methods for 3D Tumor Generation

Over the recent years, several types of 3D tumor models have been established to generate spheroids in oncology. The main 3D culture models of cancer include multicellular tumor spheroids (MCTS), tumor tissue explant, and tumor on a chip [19]. This section briefly describes the methods used to generate a multicellular tumor spheroids model, which is one of the predominantly utilized 3D tumor models. MCTs are aggregates of tumor cells that resemble spheres and are generally cultivated in suspension or embedded gels using 3D culture methods [31]. This model can partly simulate the avascular layers (proliferation zone, quiescent zone, and necrotic zone) of solid tumors. It also has phenotypic characteristics that closely resemble the cellular microenvironment in the cancerous tissue to a great extent [10]. For instance, the unevenly diffused and distribution of oxygen and nutrient gradients within larger MCTS (critical size, 400 µm) often result in the formation of distinct layers, similar to those in poorly vascularized tumors [10,31]. Thus, MTCS have become prevalent because of the overall similarities between MCTS and tumors with respect to morphology, distinct metabolic and proliferation gradients [31]. MCTS can be exploited in various experimental research studies on tumor-specific processes such as angiogenesis, invasion, and metastasis, as well as assessment of responses to various therapies such as PDT and underlying mechanisms [31]. Several simple and reproducible methods have been successfully utilized to generate MCTs, which can be categorized into two distinguishable groups: scaffold-based and scaffold-free cell techniques [10]. The scaffold-free 3D cell culture method has been commonly used to produce MCTS and is comprised of techniques such as liquid overlay, the hanging drop, and bioreactor/agitation-based methods, such as shaker flasks and spinner vessels as well as hydrogels and magnetic levitation method [10,12].

### 3.1. Hanging Drop Method

The hanging drop technique is a simple scaffold-free approach in which small droplets of the cell suspension are placed on a reversed upside-down culture lid [32]. This method involves the action of surface tension that anchors the drop of cell suspension as a hanging drop on the inverted lid [16]. Consequently, spheroids form as droplets owing to the gravity force that concentrates the cell suspension at the bottom tip [16]. Generally, the spheroids formed are tightly packed, rather than loose cell aggregates [12]. In addition to the simplicity and cost-effectiveness, this method has other advantages. This technique can form spheroids of consistent sizes and morphologies, which can be controlled by the volume of the drop or density of cell seeding suspension [32]. In addition, this method applies to a variety of cell lines and enables the creation of co-cultures with various cell types [16]. Studies reported that 3D spheroids produced by using the hanging drop technique have 100% reproducibility [12]. Nonetheless, the hanging drop protocol is one of the most time consuming and labor-intensive techniques in terms of maintaining spheroids and changing of spent culture medium, without disturbing the culture spheroid [10,12]. Another downside associated with this protocol is the inability to maintain long-term spheroids due to the generally limited volume of the seeding suspension that cannot provide sufficient nutrients [33]. Recently, studies have developed several tools such as commercial 384-well droplet suspension plates and bioassay plates to surpass some of these limitations and enhance the efficiency of spheroid formation [10,12,33].

### 3.2. Liquid Overlay Method

This technique is a simple method that prevents cells from attachment and matrix deposition on modified non-adherent culture surfaces such as well plates, flasks, or dishes [32,33]. The culture surface is generally coated with a thin layer of non-adherent inert substrates, such as agarose, agar, Matrigel, or poly(2-hydroxyethyl methacrylate), which forces the cells to remain afloat in the culture medium and possibly inhibit cell attachment to the surface of the culture vessels [10,12]. As a result of cell attachment interruption on the surface, the liquid overlay culture system facilitates an increase in cell–cell interaction and promotes cell aggregation, leading to the spontaneous formation of spheroids above the non-adherent surface [32]. For example, a recent study by Schnieder et al. [34] produced HeLa MCTS by the liquid overlay method based on agarose-coated 96-well plates to investigate the phototoxicity of tetraplatinated (metallo) porphyrin-based PS. The study reported a phototoxic index (PI) of 6030 in HeLa cells [34]. Similarly, Karges et al. [35] cultured HeLa MCTS using the liquid overlay technique and evaluated the phototoxic effect of Ru(II) polypyridine complexes with (E,E′)-4,4′-bisstyryl-2,2′-bipyridine ligand [35]. Upon irradiation, the complexes generated ^1^O_2_, causing a phototoxic effect via apoptosis and paraptosis pathways in MCTS [35]. This type of cell culturing technique is cost-effective, facilitates high reproducibility with no additional material or specific equipment, and enables co-culturing of different cell types [12]. However, the size and number of spheroids generated with liquid overlay non-adherent surface culture methods are usually difficult to control [10].

### 3.3. Bioreactor-Based 3D Culture Method

The four most common types of bioreactors used for 3D spheroid production include spinner flask bioreactors, rotational culture systems, perfusion systems, and mechanical force systems [12]. The bioreactor-based 3D culture technique generally involves filling a chamber with a cell suspension cell culture medium and then continuously agitating either by gently stirring, rotating, or pumping media through a scaffold, to induce non-adherent conditions [12,17]. Spinner cultures serve to distribute oxygen and nutrients evenly, excrete metabolic waste, and provide uniformity of the physical and chemical factors within the bioreactors, which are in favor of spheroids formation [12,16]. This method is suitable for the extensive production of biomolecules, such as antibodies or growth factors, as well as intensive cell expansion [12]. The technology of microfluidic involves the cultivation of cells in perfused hollow microchannels, allowing the distribution of oxygen and nutrients and the excretion of waste products [10,36]. Microfluidic devices, such as organ chips are made up of optically clear plastic, glass, or flexible polymers, such as polydimethylsiloxane (PDMS) [36]. As an advantage, this system can mimic in vivo organ-level physiology and pathophysiology by recreating tissue-level and organ-level structures and functions in vitro [36]. Even though bioreactor-based 3D culture models provide greater control on spheroid generation and reproducibility, this technique still relies on expensive instruments, and the produced spheroids generally have poor uniformity in size and shape [37].

### 3.4. Hydrogels

A hydrogel is an aqueous medium-inflated network of physically or chemically cross-linked polymer molecule [38]. The hydrophilic structure allows for large amounts of water to be absorbed and retained [37]. They can be designed with a wide range of compositions, biophysical properties, and biological functions, allowing them to mimic many of the characteristics of natural ECMs [31,34]. Natural biomaterials composed of polysaccharide (amylose, cellulose, alginate, chitosan, or hyaluronic acid), peptide (collagen or gelatin), nucleic acid, or polyhydroxyalkanoates are used to design the 3D culture system of cancer cells [37,39]. For instance, Nii et al. [40] investigated a cancer invasion model based on interaction between cancer cells and cancer-associated fibroblasts (CAF) aggregates [40]. Nii et al. incorporated gelatin hydrogel microspheres (GM) containing pifithrin-α (PFT) of a p53 inhibitor (GM-PFT) with the CAF aggregates [40]. The study noted that GM-PFT promoted the expression of the alpha-smooth muscle actin in CAF aggregates at the high concentration of PFT [40]. Moreover, when cancer cells were co-cultured with the CAF aggregates incorporating GM-PFT, their invasion rate was significantly high compared with CAF aggregates or CAF aggregates incorporating GM [40]. This method effectively increases cell viability while it decreases cellular apoptosis [31]. Furthermore, hydrogels can deliver soluble or signaling molecules to cells and provide a conducive environment for cell growth and function [12]. However, despite several advantages presented by hydrogels, uncertainty and ambiguity in composition caused by the gelling process can result in unwanted and nonspecific cellular responses. Furthermore, pH-based gelling mechanisms can be detrimental to delicate cells [12].

### 3.5. Magnetic Levitation Method

In the magnetic levitation-based method, the cells are combined with magnetic particles and subjected to magnetic force to surmount the gravitational force, allowing them to levitate and form cellular aggregates [41]. This method uses negative magnetophoresis to resemble a weightlessness condition, unlike positive magnetophoresis, which limits weightlessness [41]. Due to the magnetic force, the mixture of magnetic particles and cells remain gravitated against gravity [42,43]. This condition causes a change in cell mass geometry and promotes cellular interactions, resulting in cell aggregation [32,42,43]. Furthermore, this method can be applied for multi-cellular co-culturing agglomeration of different cell lines [42,43]. Another advantage of this technique is that it is a fast-acting technique with high reproducibility to generate spheroid less than 16 h [10,32]. However, some researchers showed that artificially manipulating gravity can alter cellular structures and even trigger apoptosis [32].

## 4. PDT-Induced Antitumor Immune Responses

**Figure 3:**
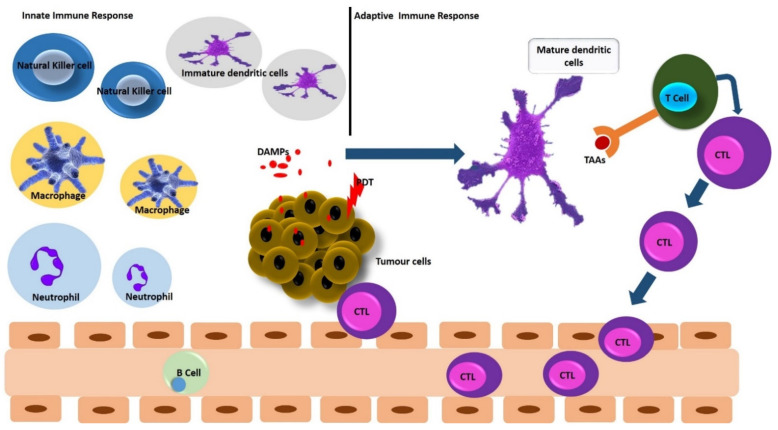
An overview of PDT-induced innate and adaptive immune responses.

Ideal anticancer treatment should induce local tumor regression and systemic antitumor immune responses capable of obliterating distant metastases with minimal toxicity to healthy tissues [44]. From this perspective, PDT holds great promise, since it provides a strong and acute inflammatory response [9]. The local inflammatory responses lead to the infiltration of neutrophils into the tumor site and the generation of pro-inflammatory factors and cytokines [44]. Meanwhile, photodamaged cells show a systemic antitumor immune response, which then activates a secondary cause of tumor cell death [45]. PDT can trigger both innate and adaptive immune responses by subjecting PDT-treated tumor cells to complementary immune cells (Figure 3) [5,46,47].

### 4.1. PDT and Innate Immune Response

The innate arm of the immune response system eliminates pathogenic agents by phagocytes (macrophages, neutrophils, and dendritic cells (DCs), the complement cascade, and natural killer (NK) cells [6]. Following an acute inflammatory response, PDT-induced activation of the innate immune system involves the release of cytokines, complement activation, infiltration, and activation of innate immune cells [2,9]. Then, post-treatment, the oxidative stress triggered by PDT leads to extended tumor tissue destruction [46]. Damaged tumor cells would exhibit damage-associated molecular patterns (DAMPs) or secrete DAMPs into the extracellular matrix [2,6,9]. Those DAMPs serve as harmful signals and can be recognized by antigen-presenting cells (mainly the DCs) [6]. DCs ingest and process tumor-associated antigens (TAAs) and present TAA-derived peptides to effector T cells, thereby coordinating an antitumor adaptive immunity, which could confer a prolonged systemic tumor immune control [9,30,32].

### 4.2. PDT and Adaptive Immune Response

The initiation of acute inflammation by PDT attracts neutrophils to the irradiated tumor area and secretes chemokines and granule proteins to promote DCs maturation and activation [44]. Activated DCs can stimulate naïve T cells to transform into cytotoxic tumor-specific T lymphocytes (CTLs) and antigens that can stimulate B cells to produce antibodies [4,9]. Upon exposure to DAMPs, which are released by PDT-damaged or dying cells, DCs can transit to a mature state and migrate to lymph nodes, which consequently present TAA-derived peptides to naïve T cells and generate CTLs to attack and obliterate residual cancer cells [6,46,47]. The mature DCSs are characterized by the overexpression of peptide-major histocompatibility (MHC) complexes on the cell surface, prime CD4+ T helper cells and CD8+ to CTLs, and trigger an adaptive immunity [5,45,47]

## 5. Experimental Studies on Immune Responses to PDT in Cancer Treatment

Several studies demonstrated that PDT stimulates both innate and adaptive immune responses that play a major role in tumor eradication [47]. Table 2 summarizes recent studies exploring the effect of PDT on immune responses. Studies were conducted on various cancer cell lines and experimental models (2D monolayer, 3D tumor models, and in vivo). It can be observed from Table 2 that several studies concerning PDT-induced immune responses were performed on in vivo tumor models. According to Poggi et al. [11], innate cells such as NK cells do not exhibit similar phenotypical and functional features in xenograft models and humans. Thus, more studies are needed in 3D tumor models to better resemble the in vivo human tumor environment before proceeding to costly and time-consuming animal studies.

## 6. Clinical Studies on PDT Immune Responses in Cancer Treatment

Abdel-Hady et al. [68] provided the first evidence for PDT-induced antitumor immunity in clinical trials when they found that patients with vulval intraepithelial neoplasia (VIN) who were resistant to ALA-PDT had a greater chance of having MHC-I negative tumors and less CD8+ T cell influx at the tumor site [63]. Studies by Kabingus et al. reported an enhanced antitumor immunity when peripheral blood mononuclear (PBMCs) cells of basal cell carcinoma (BCC) showed increased tumor antigen recognition and cytokine release following PDT treatment [69]. Thong et al. [70] reported a case study of a 64-year-old patient diagnosed with multifocal angiosarcoma whose tumors regressed after receiving high-dose brachytherapy but relapsed within 1 year. PDT treatment of the recurrent tumors using fotolon noted a spontaneous remission of the untreated tumors [70]. The biopsies of the untreated tumors revealed an increased accumulation of CD8 T cells [70]. Another study by Morrison et al. [71] investigated PDT-induced immunity on nine breast cancer patients that failed surgical excision and radiotherapy [66]. Morrison et al. noted that 67% of patients completely or partially responded to continuous low irradiance PDT (CLIPT), while 22% of patients showed a remarkable regression of tumors distant from the treatment field [71]. PDT has been reported to circumvent immunosuppression, through attenuation of Tregs in patients diagnosed with invasive esophageal squamous cell carcinoma (ESCC) [72].

## 7. Enhancing PDT-Induced Antitumor Immune Responses

### 7.1. Intracellular Accumulation of PSs

PDT-induced cell death depends on the intracellular localization and binding sites of the PSs [73]. Photoexcitation of a mitochondrion-localized PS triggers the release of cytochrome C, which in turn activates apoptosis caspase [74]. Meanwhile, photodamage to the ER causes the release of Ca^2+^, which can potentially lead to apoptosis [75,76]. PDT can also cause subcellular organelle-specific stress, since subcellular organelle-dependent oxidative stress is linked to signaling pathways in immunogenic cell death [77,78]. Therefore, by formulating PSs with a high affinity to specific subcellular organelles, it may be possible to control both the PDT process and antitumor immune response in tumor cells to improve therapeutic efficacy.

### 7.2. ER-Targeted PDT

The ER is a vital organelle that performs several essential cellular processes and can influence cancer pathogenesis [75]. ER stress is the key initiator of intracellular signaling pathways that regulate immunogenic cell death (ICD) [79,80]). It was reported that ER is the accumulation site for PS drugs such as hypericin or meta-Tetra(hydroxyphenyl)chlorin (mTHPC) and can cause an extensive ROS-based ER stress upon photoactivation [81,82]. Such ER-accumulated PSs not only exert ROS-induced cellular destruction but also can cause ICD [81]. Thus, ER plays a key role in promoting PDT efficacy via direct effects on cancer cells and indirect effects on immunity [75].

However, one major problem that could potentially hamper the application of these PSs is their inherent low absorbance wavelength that cannot reach deep-seated cancer tissues [83]. As a result, in vivo PDT treatment using hypericin or mTHPC can only be applied for superficial tumor tissue. To fully explore their ability to eradicate deep tumor tissue, it would be ideal to conserve their ER localization and amplify their light absorption wavelength to the optimum near-infrared region through different derivatives [4]. In recent years, strides have been made to develop ICD inducers that can directly and effectively trigger ER stress [84]. Studies by Li et al. [84] developed a nanocomposite that consisted of ER-targeting pardaxin (FAL) peptides functionalized with indocyanine green (ICG) conjugated-hollow gold nanospheres (FAL-ICG-HAuNS), together with an oxygen-delivering hemoglobin (Hb) liposome (FAL-Hb lipo), which was designed to counteract hypoxia. Li et al. reported that light irradiation and the nanocomposite triggered robust ER stress and calreticulin (CRT) exposure on the surface, which stimulated dendritic cells [84]. 

### 7.3. Mitochondria Targeted PDT

Mitochondria are vital subcellular organelles that undertake critical roles in metabolism, including cellular proliferation. Their actions are complexly interlinked with signaling pathways and the apoptotic process [85]. Thus, great efforts have been made in developing mitochondria-targeted PSs to enhance PDT efficacy and improve cancer treatment outcomes. Yang et al. developed mitochondria-targeting gold nanoparticles combined with triphenylphosphonium (TPP) to increase 5-ALA PS cellular uptake to allow for enhanced ROS formation and improved the selective photodamage of breast cancer cells in PDT [86]. Xu et al. [87] investigated a dual-targeting nanophotosensitizer consisting of cationic porphyrin derivative (MitoTPP) with the polyethylene glycol (PEG)-functionalized and folic acid-modified nanographene oxide (NGO) that overexpressed folate receptor and subsequently localize in mitochondria [81]. Upon photoactivation, the released MitoTPP molecule produced cytotoxic singlet oxygen and induce enhanced oxidative stress in cells [87]. Soler et al. [88] demonstrated that photoactivated silicon phthalocyanine (Pc4) accumulated in mitochondria triggered apoptosis on activated CD3+ T cells that may be used in targeting T cell-related skin disorders [82].

In addition to improved ROS generation, PDT-triggered mitochondrial apoptosis stimulates signal transduction pathways, which also promotes immune responses [88]. Studies by Marrache et al. [89] devised a mitochondria-targeted-nanoparticle (NP)-based delivery system to deliver mitochondria-acting PS for the production of tumor cell antigens, which could consequently activate DCs in vivo for possible immune response [83]. Marrache et al. reported that the nanophotosensitizer resulted in increased levels of IL-18 secretions by breast cancer cells upon photoactivation, which in turn facilitated the production of interferon-gamma (IFN-γ) of activated DCs [89]. Therefore, the study suggested that mitochondria target-specific PSs could be a novel strategy for producing effective tumor cells antigens and boosting the host immune responses post PDT treatment [89].

### 7.4. Application of Nanotechnology

Nanomedicine is a rapidly evolving field that is transforming cancer diagnosis and treatment [90]. Nanoparticles (NPs) can be broadly defined as materials that have at least one dimension (1–100 nm) in the nanoscale regime, which thus provide unique chemical and physical properties [91,92]. NPs can overcome the current challenges of PDT and have emerged as a unique approach to improve the therapeutic efficacy of PDT [93]. Since NPs are hydrophilic, they can significantly enhance the solubility of conventional PSs and their cellular uptake [1]. NP-loaded PSs can passively accumulate in tumors due to enhanced permeability and retention (EPR), which is attributed to the leaky tumor vasculature and impaired lymphatic drainage of tumor tissues [94]. Moreover, the targeted delivery of PSs can be significantly enhanced by immobilizing targeting moieties on the surface of NPs, such as antibodies, aptamers, and peptides [95]. An active targeting NPs-PS system can increase the bioavailability of PSs in the affected area while minimizing unwanted side effects of PS drugs to adjacent healthy tissues [95].

The application of nanotechnology has also provided many novel strategies for delivering multiple immunostimulatory agents in a well-coordinated and targeted manner [96,97,98]. In addition, NP-mediated systems can trigger apoptosis for the release of internal antigens or stimulate the production of cytokines [96,97,98]. Aldinucci et al. [99] incorporated DCs with carbon NPs, which resulted in the differentiation and activation of DCs at a low immunogenic profile [93]. Xiang et al. [100] developed antigen-loaded upconversion NPs (UCNPs) to track and induce the maturation of DCs in vivo as well as stimulate cytokine release. Xiang et al. reported that UCNPs enhanced T cell proliferation, IFN-γ generation, and cytotoxic T lymphocyte (CTL)-mediated responses [100]. Moreover, UCNPs exhibited photosensitizing properties and can be photoactivated using near-infrared light [100].

NPs are ideal PS delivery platforms and immunostimulatory agents due to their tunable size and surface chemistry, which allows for multiple modifications with various ligands, making them suitable for intracellular targeting [101]. In this regard, nanobioconjugates consisting of a PS and an antigen can be further functionalized with mitochondria target-specific entities to concentrate therapeutic drugs in the mitochondria for improved PDT efficacy and desirable immune responses [101]. Thus, NPs will serve as a powerful tool for combining the primary cell killing function of PDT with the secondary cell killing function of PDT-induced immunity [102].

## 8. Experimental Models for Evaluation of PDT-Induced Antitumor Immune Responses

To bridge the gap between preclinical studies and clinical trials, it remains imperative to evaluate the potency of PDT-induced immunity using tumor models that can readily recapitulate human response [33]. Since 2D monolayer cell cultures do not resemble the intrinsic 3D human tissue architecture, significant discrepancies have been noted when replicating experiments from 2D monolayer cultures to in vivo conditions [10]. Therefore, xenografts that readily integrate the immunity system are widely used platforms for cancer immunology research as well as evaluating PDT-induced cytotoxicity [103,104]. Human tumor cells are typically inoculated in immunocompromised animals lacking essential immune factors to avoid host immune system rejection and achieve desired tumorigenesis [103,104]. Thus, current preclinical studies use immune-suppressed mouse models as a reliable means of studying PDT-induced antitumor immunity in order to investigate important interactions between tumors and the immune system as well as their response to novel modalities such as PDT. Although these animal studies improve the success rates of anticancer agents in clinical trials, murine models poorly reflect the physiological conditions in humans as they inherently contain non-human host cells [10,104]. Furthermore, besides the high cost and ethical complexities, the rate of tumor growth in immunocompromised xenograft models is faster than in natural human tumors and thereby responding better to anticancer drugs [10,11].

In this sense, the development of 3D tumor models that closely resemble human tumors and the microenvironment can add a significant value in predicting the clinical efficacy of PDT and PDT-induced immune responses in vivo [105]. Following extensive research into the role of the extracellular matrix (ECM) in cellular signaling kinetics, it became evident that 3D tumor models are an excellent approach to mimic the intrinsic tumor microenvironment. In these models, cells are cultivated in a spatial manner to mimic the native tumor microenvironment by providing cell–cell and cell–ECM interactions [10]. Hopefully, 3D tumor models could reduce or, potentially, supersede the use of xenograft models, thereby circumventing the ethical and cost issues [106,107]. Since PDT can trigger strong antitumor immune responses, great efforts have been made to potentiate this effect by stimulating various immune components [108]. This can be achieved by coupling PDT with immunostimulants to increase leukocyte infiltration, improve tumor antigen presentation, upregulate T-cell activation, and attenuate immunosuppressants [108].

### 8.1. Immunomodulation

Although several pieces of evidence have established the capacity for PDT to obliterate the primary tumors and provide durable antitumor immune responses, some limitations were reported [109,110] due to the fact that tumors are multifaceted and exhibit different immunogenicity as reflected by infiltrated immune cells [111]. Another obstacle to PDT-induced immunity is the rapid release of immunosuppressive factors at the tumor site [111], which typically occurs in metastatic stages [112]. Many strategies were explored to boost the immune system and counteract the release of immunosuppressive factors, thereby enhancing the overall efficacy and durability of PDT-induced immune responses against cancer.

#### 8.1.1. Immunostimulants

Several adjuvants have been developed to enhance anticancer agents: Toll-like receptor (TLR) agonists, such as Bacillus Calmette–Geurin (BCG, TLR-2/4), imiquimod (TLR-7), and CPG oligodeoxynucleotide (CpG ODN, TLR), stimulate innate and adaptive immune responses [113]. They interact with pattern recognition receptors (PRRs) on immune cells, which increases the antigen delivery to APCs, as well as stimulating the release of the immunomodulatory cytokine [113]. Studies showed that the combination of PDT and BCG caused tumor regression in mice, regardless of the type of PS that was used, including photofrin, benzoporphyrin derivative, foscan, mono-l-aspartyl-chlorin e6, lutetium texaphyrin, or zinc phthalocyanine [114]. Intriguingly, BCG combined with photoactivated photofrin increased the number of memory T lymphocyte subsets at tumor lymph nodes when compared to photofrin treatment alone [114]. Studies by Bae et al. reported that radachlorin combined PDT treatment with CpG ODN triggered a robust antitumor immune response, which stimulated the production of tumor-specific antibodies and cytotoxic T cell responses [115]. In addition, photoactivated verteporfin in conjunction with CpG caused tumor regression and increased potency compared to either treatment alone [116]. Topically administered PDT with imiquimod cream was shown to be more effective on invasive squamous cell carcinoma than either treatment alone in both xenograft models and humans [117]. Zymosan, a TLR-2 agonist, demonstrated enhanced tumor inhibitory effect post-PDT as well as increased C3 complement levels [118]. Other TRL agonists, such as mycobacterium cell wall extract (MCWE) in combination with various PSs, improved PDT therapeutic efficacy [119]. In addition, new immunoadjuvants, such as the semisynthetic biopolymer N-dihydrogalactochitosan, have been developed in recent years in order to prolong antitumor responses and enhance PDT-induced cell kill effects [120].

Tumor ICD is initiated by the release of DAMPs, such as CRT, HSP70, HMGB1, or ATP, which can promote immune maturation and activation at the targeted tumor region through their interactions with PRRs [121]. It was shown that recombinant CRT can enhance the therapeutic effect of PDT and/or PDT-related anticancer agents when administered peritumorally [121].

#### 8.1.2. Blocking Immunocompromising (Cellular) Factors

The tumor microenvironment (TME) has a strong immunosuppressive effect, which is the main reason behind the low efficiency of cancer therapies that act by stimulating immunity against cancer [8]. TME is characterized by the upregulation of inhibitory factors that counteract immune activation as well as the promotion of cell proliferation and infiltration of immunosuppressive cells [8]. Therefore, some strategies can be used to relieve the suppression of the immune system and enhance the systemic antitumor immune response of PDT [122]; particularly, immune checkpoints inhibitors, such as programmed death-ligand 1 (anti-PD-L1) and anti-T-lymphocyte-associated protein 4 (anti-CTLA-4), have revolutionized for the treatment of cancer [123]. The blockade of PD-L1 or CTLA-4 conserves the anticancer effects of lymphocytes to provide remarkable synergistic effect with PDT [124,125,126,127]. Furthermore, PS combined with targeting moieties such as antibodies in targeted PDT has revealed a synergistic effect, resulting in DCs maturation and activation, T lymphocytes infiltration, abscopal effects, and immunologic memory [128,129]. Another potent suppression of PDT-mediated antitumor immune response results from Tregs and myeloid-derived suppressive cells (MDSCs) [8]. Therefore, inhibiting these immunomodulatory cells provides an additional promising approach to improve immune tumor control [8]. Studies by Reginato et al. [130] reported a drastic tumor regression and increased survival upon the specific depletion of Tregs using a low concentration of cyclophosphamide before PDT treatment. In addition, the depletion of MDSCs through GR1 blocking antibody improved the antitumor effect of PDT [131]. However, immediate administration of anti-GR1 showed a decrease in potency when compared to 1 h administration after PDT irradiation. The discrepancies in the therapeutic efficacy could be attributed to the undesirable neutrophil depletion by anti-CR1 during the acute phase of PDT, where neutrophils play a key role in immune responses stimulation [131]. Additionally, soluble mediators released by TME such as tumor-derived beta (TGF-β) and prostaglandin E2 (PGE2) also attenuate antitumor immune responses [132]. Thus, targeting these factors can improve the PDT-induced antitumor immune response [132].

#### 8.1.3. Recognition of Tumor-Associated Antigens (TAA)

Tumor cells can be differentiated from normal cells by their overexpression of tumor-specific antigens [133]. The initiation of the systemic adaptive immune response following PDT relies on the maturation of DCs, recognition of tumor antigens by DCs, and the activation of CTL [134]. The immune effects of PDT strongly depend on the degree of antigen presentation and recognition of tumor antigens by immune cells [109]. It was noted that oxidative stress can accelerate the expression and release of antigens [109]. In reality, most human tumors are less immunogenic, which drastically affects PDT efficacy and immunogenic effects in the long term [8,134]. In addition, tumor cells can evade immune system barriers through the downregulation of MHC1 molecules or loss of tumor antigen expression [134]. Various approaches were explored to accelerate the tumor antigen expression or presentation by APCs. One of these strategies is to modify the genetic aspect of tumor cells to improve their immunogenicity. Wachowska et al. [135] demonstrated that PDT can initiate robust antigen-specific antitumor immunity against tumors expressing the P1A antigen, which is a form of TAA. They incorporated PDT with a clinically approved epigenetic agent 5-aza-20-deoxycytidine that can provide the expression of P1A antigen in various tumor cells. According to their findings, inducing P1A expression through epigenetic modification improved PDT antitumor immune responses and stimulated immunological memory in tumor-bearing mice [135].

## 9. Conclusions and Future Perspectives

In recent years, PDT has gained a great deal of attention due to its ability to eradicate primary tumors. It generates systemic and long-lasting antitumor responses that combat metastases and tumor recurrence. Although clinical evidence is very scarce, numerous preclinical studies showed that PDT modality could potentially become a potent therapeutic option for cancer treatment. Therefore, researchers have explored several approaches aimed at overcoming the immunotolerance in treated tumors, attenuating the immunosuppressive TME and establishing a robust and systemic adaptive immune response that can obliterate distant tumor lesions. Combinational therapies that synergize with the immunostimulant role of PDT may pave a way for the successful utilization of PDT as a mainstay treatment in the clinical arena. Additionally, the improved intracellular delivery of PS and immunostimulatory agent integrated with nanotechnology is a great stride forward in enhancing the efficacy of PDT and may endorse the rational design of a PDT regimen. A physiologically relevant tumor model that closely resembles the 3D architecture and functional properties of the solid human tumor is essential for the evaluation of tumor mass and immune system interaction as well as PDT-triggered antitumor immunity. Currently, the majority of PDT and immunity experiments have been performed on traditional 2D monolayer cell cultures. Several lines of evidence suggest that promising drug candidates fail clinical trials, thereby hampering the discovery of potent therapeutics. This is because the cellular environment of 2D cell cultures does not resemble that of real tumors. Thus, to validate in vitro data, in vivo studies have been intensely investigated, using animal models that may closely reflect what happens in humans. As a matter of fact, tumors are artificially generated within a short period in these xenografts’ models. Furthermore, these animals are immunosuppressed and contain immune cells that differ from their human counterparts. To minimize the number of animals used, and perhaps replace these costly and cumbersome animal studies, 3D tumor models have been developed. Three-dimensional (3D) cell cultures may bridge between preclinical and clinical studies, since they are scientifically accurate and simulate different aspects of human tumors. Thus, spheroids have a significant value in predicting the clinical efficacy of anticancer drugs. The overall findings of this review concluded that very few studies in relation to PDT-induced antitumor immunity were conducted within 3D tumor models (Table 2). Thus, this warrants further investigation in 3D tumor models to bridge the gap between in vitro and in vivo studies for improved preclinical phases and successful clinical trials outcomes.

## Figures and Tables

**Table 1:** Characteristics of 2D versus 3D cell culture models.

Characteristic	2D	3D	Refs.
In vivo-like	Poor resemblance of the 3D architecture of tumor tissue	Mimic the 3D structure of in vivo tumor tissues	[21]
Proliferation	Cells grown in monolayers proliferate faster than in 3D tumor models	A relatively slow proliferation rate is similar to that of human tumor cells	[22]
Polarity	Partial polarization	A precise portrayal of cell polarization	[23]
Morphology	Flat and sheet-like cells with a stretched appearance	Form aggregated cells.	[24]
Rigidity	Strong rigid (about 3 × 10^9^ Pascals)	Less rigid (>4000 Pascals)	[25]
Cellular interactions	Limited cellular interactions and cellular extracellular matrix	Exhibit cellular interactions and cell-extracellular matrix-like solid tumors	[16]
Gene/protein expression	Alterations in gene expression, mRNA splicing, topology, and biochemistry of cells, often show discrepancies in gene/protein levels when compared to in vivo models	Genes and protein expressions in solid tumors pertinently resemble 3D tumor models	[26,27]
Response to therapeutics	Monolayer cell cultures are more susceptible to drugs than human tumors	Tumor cells in 3D cultures exhibit drug resistance characteristics similar to those observed in vivo human tumors	[16,26]
Culture formation	Takes minutes–hours	Take hours–days	[28]
Culture quality	Good performance, reproducible, long-term culture, ease of interpretation, and culture simplicity	Poor performance and reproducibility, difficult interpretation, and cultures	
Access to growth factors	Constant exposure of cells to oxygen, nutrients, metabolites, and signaling molecules (as opposed to in vivo)	Limited distribution of oxygen, nutrients, metabolites, and signaling molecules (similar to in vivo)	[16,29]
Cost of maintenance	Cost-effective, abundant commercially available tests and media	Costly, laborious, and lack of commercially available tests	[30]

**Table 2:** Experimental studies on immune responses to PDT in cancer treatment.

Generation	PS	Localization	Cell Line	Tumor Model	Animal Species	Hallmarks of Immunogenic Cell Death (ICD) In Vitro	Hallmarks of ICD In Vivo	Refs.
1st	Photofrin	Mitochondria, cellular membrane	Lewis lung carcinoma (LLC) cells	2D monolayer cell culture and in vivo	C57BL/6 mice	PDT-treated LLC increased the expression of high-mobility group box-1 (HMGB1) protein in macrophages	PDT accelerated the expression of calreticulin (CRT) and (HMGB1) protein in LLC tumors in vivo.	[48]
			AB12 Mesothelioma	in vivo	Balb/c mice	Localized neutrophil function at 1 h and then drops at 4 h. Increased infiltration of neutrophils at the treated at 24 h	N/A	[49]
2nd	OR141	Endoplasmic reticulum (ER)	AB12 Mesothelioma	2D monolayer cell culture, in vivo	Balb/c mice	Maturation of DCs (increased levels of CD80, CD86, CD40 and MHC)	PDT-OR141 showed robust CD8+ and CD4+T responses with increased proliferation, cytotoxic reactions and increased production of interferon-gamma (IFNγ).	[50]
			Mouse SCC7, Human A431 squamous cell carcinoma cells and mouse B16 melanoma cells	2D monolayer cell culture	N/A	Maturation of DCs (increased expression of MHC-ll+, CD80+ and CD86+)	N/A	[51]
	Hypericin	ER	T25 human bladder carcinoma cells	2D monolayer cell culture	N/A	Maturation of DCs (increased CD80, CD83, CD86, and MCH ll) and functional stimulation (increased NO and L-1β, absent IL-10)	N/A	[52]
			GL261 glioma cells	2D monolayer cell culture and in vivo	C57BL/6 mice	Maturation of DCs (elevated levels of CD80, CD86, CD40 and MHC I)	PDT stimulated the accumulation of T-lymphocytes (CD3+, CD4+ and CD8+), TH1 cells, CTLs and TH17 cells at the treated sites	[53]
	Rose bengal (RB)	N/A	CT26 colorectal carcinoma cell line	2D monolayer cell culture and in vivo	Balb/c mice	Upregulation of CRT expression A dose-dependent decrease in ATP Increased extracellular content of HMGB1 Increased expression of HSP90	PDT-RB stimulated the expression of CRT and HSP90 on tumor cells and the release of HMGB1.	[54]
	5-Aminolevulinic acid (5-ALA)	ER	PECA squamous cell carcinoma cell line	2D monolayer cell culture and in vivo	SKH-1 mice	Maturation of DCs (upregulation of MHC-II, DC80, and CD86) and increased production of IFN-γ and IL-12	PDT upregulated expression of CD80, CD86, and MHC-II and induced T cell proliferation	[55]
			PECA squamous cell carcinoma cell line	2D monolayer cell culture and in vivo	SKH-1 mice	PDT improved the expression of CRT, HSP70, and HMGB1	Simulated phenotypic maturation (increased MHCII, CD80, and CD86)	[56]
			Glioblastoma (GB) cell lines U87 and U251	3D tumor spheroids	N/A	Maturation of DCs (increased levels of CD40, CD80, CD83, and CD86)	N/A	[57]
			PECA squamous cell carcinoma	in vivo	SKH-1 mice	N/A	Infiltration of T-lymphocytes (CD4+/CD8+) at 7 days	[58]
	Redaporfin	ER and Golgi apparatus GA	CT26 colorectal carcinoma cell line	in vivo	Balb/c mice	N/A	PDT resulted in a strong neutrophilia (2–24 h), the systemic elevation of IL-6 (24 h), increased number of CD4+ and CD8+ T cells, as well as increased production of IFN-γ or CD69+.	[59]
	Photodithazine	ER and Golgi apparatus	GL261 murine glioma, MCA205 murine sarcoma	2D monolayer cell culture and in vivo	C57BL/6J	Maturation of DCs (increased CD40, CD86, and MHC II) and increase in IL-6	PDT stimulated the release of calreticulin, HMGB1 and ATP, which activated the production of IL-6.	[60]
3rd	Core–shell gold nanocage coated with manganese dioxide and hyaluronic acid (AMH)	Hyaluronic acid targets CD44-overexpressed on the plasma membrane of CT26 cancer cells	CT26 colorectal carcinoma cell line	2D monolayer cell culture	N/A	Maturation of DCs (upregulation of CD83, CD86, MHC II)	N/A	[61]
	Cetuximab-IR700	Cetuximab binds to HER1-overexpressed on the plasma membrane of cancer cells	A431 human epidermoid carcinoma	2D monolayer cell culture and in vivo	Athymic nude mice	Maturation of DCs (increased expression of CD80, CD86, MHC II) and increased production of IL-12	Increased population of CD86+ DCs, CD11c, CD205, and MHC II positive cells.	[62]
	Core–shell gold nanocage@manganese dioxide (AuNC@MnO_2_, AM)	N/A	4T1 murine mammary carcinoma	2D monolayer cell culture and in vivo	Balb/c mice	Maturation of DCs (overexpression of CD83, and CD86) and increased production of IL-12	PDT resulted in intratumoral increase in CD11c+CD86+ and CD11c+CD83+ DCs, as well as increased NK cells and CD8+ and CD4+	[63]
	Hybrid protein oxygen nanocarrier with chlorin e6 encapsulated (C@HPOC)	N/A	4T1 murine mammary carcinoma	2D monolayer cell culture and in vivo	Balb/c mice	Maturation of DCs (increased CD86 and MHC II)	An influx of NK cells, T cells (CD8+ CD4+) at the tumor site, and maturation of DCs.	[64]
	Benzoporphyrin Derivative nanoconjugates modified with cetuximab, transferrin and trastuzumab	Cetuximab binds with anti-EGFR mAb, transferrin with glycoprotein and trastuzumab binds with anti-HER-2 mAb	PDAC Pancreatic cancer cells	3D tumor spheroids	N/A	PDT triggered the expression of heat shock-related proteins (Hsp60, Hsp70), caltreticulin and high-mobility group box 1 in light intensity and time-dependent manner. A similar trend was observed in CD4+ and CD8+ T cells antitumor reactivity by upregulating CD107a and IFN-γ	N/A	[65]
	5-ALAdoamine) dendrimers generation two (PAMAM-G2)	Endo-lysosomes and mitochondria	B16 and A375 metastatic melanoma cells	in vivo	C57BL6J mice	N/A	Prevented tumor metastases. Inhibited tumor-recurrence. Infiltration of CD4+ CD8+ T cells at the tumor region, predominately central memory T cells (CD44^high^ CD62L^high^). Insignificant change of CD3+ T cells in the spleen. Increased levels of TNF-α and IFN-γ in serum. Maintained immune balance and prolonged recurrence-free survival	[66]
	Aluminum-phthalocyanine nanoemulsion (AlPcNE)	N/A	B16F10 cells	in vivo	C57BL/6 mice	N/A	PDT-AlPcNE induced a significant release of HMGB1 and ATP as well as the expression of CRT on the plasma membrane	[67]

## Data Availability

The datasets generated during and/or analyzed during the current study is available from the corresponding author upon request.

## Abbreviations

2D	Two-dimensional
3D	Three-dimensional
APCs	Antigen-presenting cells
BCC	Basal cell carcinoma
BCG	Bacillus Calmette–Geurin
CLIPT	Continuous low irradiance PDT (CLIPT)
CRT	Calreticulin
CTL	Cytotoxic tumor-specific T lymphocytes
DAMPs	Damage-associated molecular patterns
DCs	Dendritic cells
ECM	Extracellular matrix
EPR	Enhanced permeability and retention
ER	Endoplasmic reticulum
ESCC	Esophageal squamous cell carcinoma
HMGB1	High-mobility group
Hsp60	Heat shock-related proteins
ICD	Immunogenic cell death
IL	Interleukins
IFNγ	Interferon-gamma
LLC	Lewis lung carcinoma
MCTS	Multicellular tumor spheroids
MCWE	Mycobacterium cell wall extract
MDSCs	Myeloid-derived suppressive cells
MHC	Major histocompatibility complex
NK	Natural killer cells
NP	Nanoparticles
PBMCs	Peripheral blood mononuclear
PDT	Photodynamic therapy
PGE2	Prostaglandin E2
PRRs	Pattern recognition receptors
PS	Photosensitizer
ROS	Reactive oxygen species
TAA	Tumor-associated antigens
TGF-β	Transforming growth factor beta
TME	Tumor microenvironment
TNF-α	Tumor necrosis factor alpha
TPP	Triphenylphosphonium
Tregs	Regulatory T cells
TRL	Toll-like receptor
VIN	Vulval intraepithelial neoplasia
